# In-Vitro Neutralization of the Neurotoxicity of Coastal Taipan Venom by Australian Polyvalent Antivenom: The Window of Opportunity

**DOI:** 10.3390/toxins12110690

**Published:** 2020-10-31

**Authors:** Umesha Madhushani, Geoffrey K. Isbister, Theo Tasoulis, Wayne C. Hodgson, Anjana Silva

**Affiliations:** 1 Department of Parasitology, Faculty of Medicine and Allied Sciences, Rajarata University of Sri Lanka, Mihintale 50300, Sri Lanka; madhushanih.h.u@gmail.com; 2 Clinical Toxicology Research Group, University of Newcastle, Callaghan 2308, Australia; geoff.isbister@gmail.com (G.K.I.); theo.tasoulis@newcastle.edu.au (T.T.); 3 Monash Venom Group, Department of Pharmacology, Biomedical Discovery Institute, Monash University, Clayton 3800, Australia; wayne.hodgson@monash.edu

**Keywords:** taipan, paralysis, antivenom, pre-synaptic, post-synaptic, venom

## Abstract

Coastal taipan (*Oxyuranus scutellatus*) envenoming causes life-threatening neuromuscular paralysis in humans. We studied the time period during which antivenom remains effective in preventing and arresting in vitro neuromuscular block caused by taipan venom and taipoxin. Venom showed predominant pre-synaptic neurotoxicity at 3 µg/mL and post-synaptic neurotoxicity at 10 µg/mL. Pre-synaptic neurotoxicity was prevented by addition of Australian polyvalent antivenom before the venom and taipoxin and, reversed when antivenom was added 5 min after venom and taipoxin. Antivenom only partially reversed the neurotoxicity when added 15 min after venom and had no significant effect when added 30 min after venom. In contrast, post-synaptic activity was fully reversed when antivenom was added 30 min after venom. The effect of antivenom on pre-synaptic neuromuscular block was reproduced by washing the bath at similar time intervals for 3 µg/mL, but not for 10 µg/mL. We found an approximate 10–15 min time window in which antivenom can prevent pre-synaptic neuromuscular block. This time window is likely to be longer in envenomed patients due to the delay in venom absorption. Similar effectiveness of antivenom and washing with 3 µg/mL venom suggests that antivenom most likely acts by neutralizing pre-synaptic toxins before they interfere with neurotransmission inside the motor nerve terminals.

## 1. Introduction

Snake envenoming is a major health issue in the tropics [1]. Estimates suggest that up to 1.8 million envenomings and 90,000 deaths occur globally each year from snakebites [2]. Neuromuscular paralysis is a common, life-threatening clinical effect of envenoming by some cobras (genus: *Naja*), kraits (genus: *Bungarus*), coral snakes (genera: *Micrurus*, *Calliophis*), some Australasian elapids (*Oxyuranus*, *Acanthophis*, and *Notechis* species), and some viperids (*Crotalus*, *Daboia*, *Vipera* species) in humans [3,4].

Neurotoxic snake venoms possess toxins acting pre- or post-synaptically at the neuromuscular junction, resulting in neuromuscular blockade. Pre-synaptic neurotoxins (also known as β-neurotoxins) are phospholipase A_2_ toxins which enter the motor nerve terminals and diminish vesicular recycling that results in a depletion of the synaptic vesicles. Subsequently, motor nerve terminals are damaged structurally resulting in denervation of the muscles. This damage to the motor nerve terminal is not readily reversible and resolution of nerve function requires the regeneration of the motor nerve terminal [5,6,7,8]. Post-synaptic neurotoxins (also known as α-neurotoxins) are three-finger toxins that competitively antagonise the nicotinic acetylcholine receptors (nAChR) at the motor end-plate [9,10]. There are two major types of α-neurotoxins, namely short-chain and long-chain α-neurotoxins, based on their structural and functional characteristics [9]. Many medically important neurotoxic snakes possess both pre- and post-synaptic neurotoxins in their venoms. Human nAChR have some resistance against snake α-neurotoxins, making them less clinically important in human envenoming [10].

The ability of antivenoms to effectively reverse snakebite neurotoxicity depends on the (a) ability of antibodies to bind with the different neurotoxins (efficacy), (b) the ability of antibodies (or antibody fragments) to neutralize the neurotoxic properties of the toxins, as well as (c) the ability of antibodies to reach the target sites (motor nerve terminal and post-synaptic membrane) [11]. In the case of pre-synaptic neurotoxins, once they enter the motor nerve terminals and initiate their action, the ability of antibodies to enter the motor nerve terminals and to reverse the pathophysiological process is questionable [4,5]. In contrast, antibodies are able to enter the neuromuscular junction and bind to the α-neurotoxins already bound to the nAChR. This accelerates the dissociation of the toxin-receptor complex, reversing neurotoxicity [10,12].

In-vitro neuromuscular preparations such as the isolated chick biventer cervicis nerve–muscle preparation and mouse phrenic nerve-hemidiaphragm are used to study the neurotoxic activity of snake venoms and the efficacy of antivenoms [4,13]. The chick biventer cervicis nerve-muscle preparation has the advantage of containing both focally- and multiply-innervated muscle fibres, which enable researchers to distinguish between pre-synaptic neuromuscular block and post-synaptic neuromuscular block [13].

Australian coastal taipan (*Oxyuranus scutellatus*) envenoming causes progressive neuromuscular paralysis in humans [14]. Taipoxin is the clinically important neurotoxin in coastal taipan venom and is a trimeric PLA_2_ presynaptic neurotoxin [15]. *O. scutellatus* venom also contains several short-chain α-neurotoxins namely, taipan toxin 1, taipan toxin 2 [16] and α-scutoxin 1 [17]. The early administration of antivenom can prevent the occurrence of paralysis and prevent the worsening of existing paralysis in taipan envenoming [14]. The time scale over which the antibodies remain effective in preventing the paralysis in taipan envenoming is essential to effective antivenom treatment.

We aimed to investigate the time during which the Australian commercial polyvalent antivenom remains effective in preventing the occurrence and reversal of already existing pre- and post-synaptic in vitro neuromuscular block caused by coastal taipan venom and taipoxin.

## 2. Results

### 2.1. Concentration-Dependent Neurotoxicity of O. scutellatus Venom

**Figure 1:**
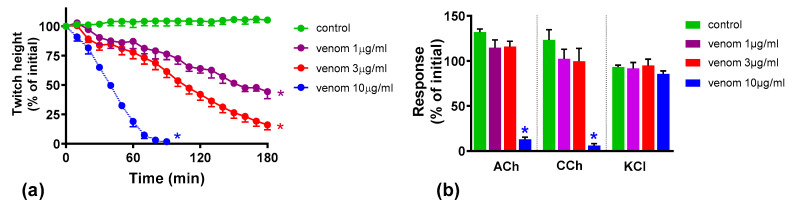
In vitro neurotoxicity of *O. scutellatus* venom: (**a**) concentration-dependent inhibition of indirect twitches in chick biventer nerve-muscle preparation by venom. (* indicates significantly different from control at the corresponding time, *p* < 0.05, one-way ANOVA followed by Tukey’s multiple comparison test, *n* = 3–5); (**b**) effect of venom on responses to exogenous agonists acetylcholine (ACh), carbachol (CCh) and KCl (* indicates significantly different from control, *p* < 0.05, one-way ANOVA followed by Tukey’s multiple comparison test, *n* = 4–5).

Venom at concentrations of 1 and 3 µg/mL inhibited indirect twitches of the chick biventer preparation by 56% and 84%, respectively, and did not abolish the responses to exogenous nicotinic receptor agonists acetylcholine (ACh) and carbachol (CCh) within 180 min, showing predominantly pre-synaptic neurotoxicity at these concentrations (Figure 1). Venom at 10 µg/mL abolished indirect twitches and the responses to ACh and CCh, but not KCl, within 90 min indicating a post-synaptic neuromuscular blockade at this concentration.

### 2.2. Effect of Australian Polyvalent Antivenom on the Pre-Synaptic Neurotoxicity by O. scutellatus Venom

**Figure 2:**
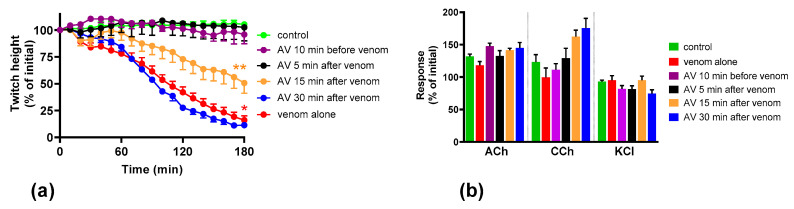
Effect of the Australian polyvalent antivenom on *O. scutellatus* venom pre-synaptic neurotoxicity: (**a**) effect of the antivenom (AV) on preventing/arresting the inhibition of indirect twitches caused by 3 µg/mL venom (* indicates significantly different from control at 180 min, ** indicates significantly different from both control and venom at 180 min, *p* < 0.05, one-way ANOVA followed by Tukey’s multiple comparison test, *n* = 4–5); (**b**) the effect of antivenom, added at different time points, on the venom-mediated response to exogenous agonists acetylcholine (ACh), carbachol (CCh) and KCl.

Venom at 3 µg/mL was used for antivenom experiments because it caused only pre-synaptic neuromuscular blockade with no evidence of post-synaptic blockade. Prior addition (10 min) of Australian polyvalent antivenom prevented the inhibition of the indirect twitches of the chick biventer nerve-muscle preparation by venom (Figure 2). Antivenom added 5 min after venom also prevented the inhibition of indirect twitches. Antivenom added 15 min after venom partially prevented the inhibition of indirect twitches of the chick biventer nerve-muscle preparation (50% of the original twitch height), indicating partial neutralization of pre-synaptic neurotoxic effects. Antivenom added 30 min after venom did not prevent the inhibition of indirect twitches by the venom (Figure 2), indicating the failure of neutralization of the pre-synaptic neurotoxic effects (Figure 2).

### 2.3. Effect of Australian Polyvalent Antivenom on Taipoxin

**Figure 3:**
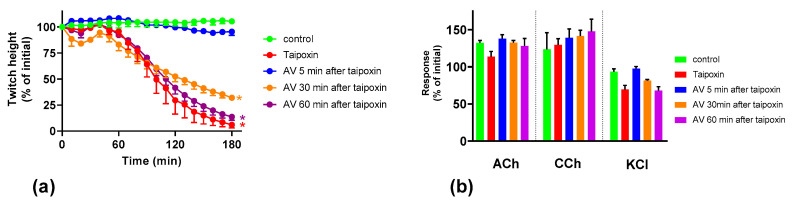
Effect of Australian polyvalent antivenom (AV) on taipoxin-mediated pre-synaptic neurotoxicity: (**a**) reversibility of the inhibition of indirect twitches caused by taipoxin (* indicates significantly different from control at 180 min, *p* < 0.05, one-way ANOVA followed by Tukey’s multiple comparison test, *n* = 3–5); (**b**) effect of taipoxin on responses to exogenous agonists acetylcholine (ACh), carbachol (CCh) and KCl.

To test the ability of the Australian polyvalent antivenom to prevent/arrest the neurotoxicity caused by taipoxin, antivenom was added 5, 30 and 60 min after the toxin (100 nM). Addition of antivenom 5 min after taipoxin fully prevented the inhibition of indirect twitches, indicating neutralization of the pre-synaptic effect of taipoxin (Figure 3). Antivenom added after 30 min and 60 min did not reverse the inhibition of indirect twitches by taipoxin (Figure 3), indicating the failure of neutralization of the pre-synaptic effect of taipoxin.

### 2.4. Effect of Washing the Neuromuscular Preparation on the Neurotoxicity of O. scutellatus Venom

**Figure 4:**
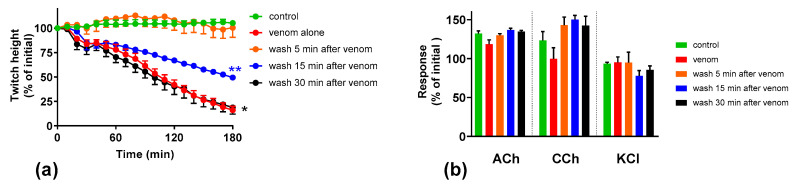
Effect of washing the preparation on *O. scutellatus* venom pre-synaptic neurotoxicity: (**a**) effect of washing on the inhibition of indirect twitches caused by venom (3 µg/mL). (* indicates significantly different from control at 180 min, ** indicates significantly different from both control and venom at 180 min, *p* < 0.05, one-way ANOVA followed by Tukey’s multiple comparison test, *n* = 4-5); (**b**) the effect of washing at different time points on the venom response to exogenous agonists acetylcholine (ACh), carbachol (CCh) and KCl.

To test the effect of the removal of the venom from the organ baths on the prevention/reversal of the neuromuscular block caused by the venom, the organ baths were washed after exposing the preparation to venom (3 µg/mL) for different time intervals. When the preparations were washed after 5 min, the venom-mediated inhibition of the indirect twitches of the chick biventer nerve-muscle preparation was fully prevented (Figure 4). When the preparations were washed after 15 min, the venom-mediated inhibition of indirect twitches was partially reversed. Washing after 30 min did not reverse the inhibition of indirect twitches of the chick biventer nerve-muscle preparation (Figure 4).

### 2.5. The Effect of Washing and Australian Polyvalent Antivenom on Neuromuscular Block Caused by O. scutellatus Venom at 10 µg/mL

**Figure 5:**
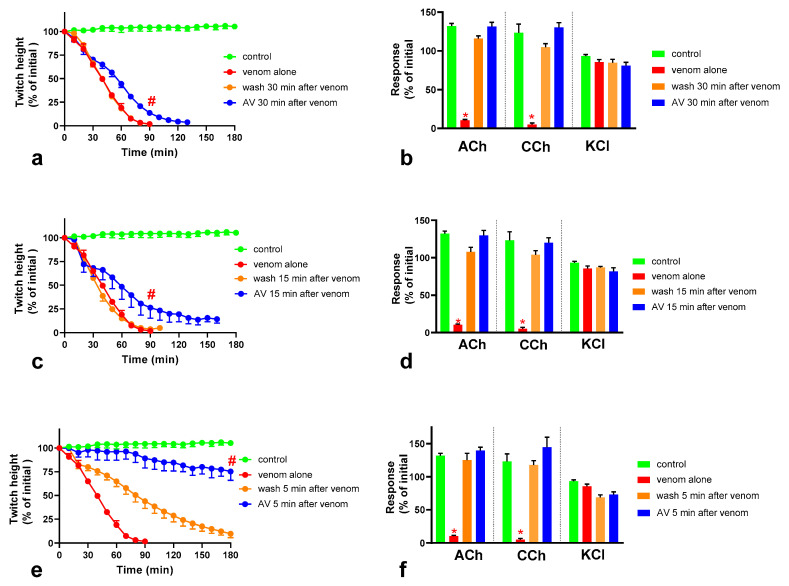
Effect of washing and Australian polyvalent antivenom (AV) on the neuromuscular block caused by 10 µg/mL *O. scutellatus* venom, when applied after different time intervals following the application of venom: the effect of washing and antivenom on the venom-mediated inhibition of indirect twitches and the response of the neuromuscular preparation to exogenous agonists acetylcholine (ACh), carbachol (CCh) and KCl when added or commenced after 30 min (**a**,**b**), 15 min (**c**,**d**) and 5 min (**e**,**f**) (# indicates the twitch height of the AV added preparations is significantly different to preparations washed at the corresponding time point, *p* < 0.05, Mann Whitney test, *n* = 3–5; * indicates significantly different from control, *p* < 0.05, one-way ANOVA followed by Tukey’s multiple comparison test, *n* = 3–5).

In this experiment, *O. scutellatus* venom was used at 10 µg/mL to cause complete post-synaptic and pre-synaptic neuromuscular blockade. Neither washing the preparation, or the addition of Australian polyvalent antivenom after 30 min exposure of the preparation to 10 µg/mL venom, prevented the venom-mediated abolishment of indirect twitches, indicating an inability to prevent the neuromuscular block caused by the venom after 30 min (Figure 5a,b). Both antivenom and washing prevented the venom-mediated abolishment of the response of the muscle to ACh and CCh, indicating a reversal of the post-synaptic, but not the pre-synaptic, neuromuscular block caused by the venom. However, at 90 min post-venom (i.e., 60 min after antivenom or washing), the remaining twitches of antivenom-treated preparations were higher compared to preparations subjected to washing. Similar observations were made when the effect of antivenom and washing after 15 min exposure of the preparations to 10 µg/mL venom was tested (Figure 5c,d). The addition of antivenom at 5 min post-venom resulted in retention of 75% of the initial twitch height after 3 h, while neutralizing both pre-and post-synaptic effects (Figure 5e,f). The washing of the preparation at 5 min post-venom prevented only the post-synaptic blockade but not the pre-synaptic blockade.

## 3. Discussion

We found that although *O. scutellatus* venom displays concentration-dependent neurotoxicity in the isolated chick biventer nerve muscle preparation, the nature of the blockade of neurotransmission changes with the concentration of venom present. A predominantly pre-synaptic action of the venom is observed at 3 µg/mL. At 10 µg/mL, there is an additional post-synaptic action of the venom observed. This indicates that the effects of the potent pre-synaptic toxin, taipoxin, occur at lower venom concentrations in the absence of post-synaptic neurotoxicity. At higher venom concentrations, the more rapidly acting post-synaptic toxins act and may mask the effects of the pre-synaptic toxin. The in-vitro pre-synaptic neurotoxicity of the venom was prevented by the addition of Australian polyvalent antivenom before the venom, or when added 5 min after the addition of venom. The antivenom only partially reversed the pre-synaptic neurotoxicity when added 15 min after venom, and was ineffective when added 30 min after the venom. In contrast, the post-synaptic activity of venom (10 µg/mL) was fully reversed even when the antivenom was added 30 min after the venom.

The exact mechanism by which pre-synaptic toxins act remains unclear. Although some authors have proposed receptors located in the pre-synaptic membrane, the existence of these receptors remains controversial [8,18,19,20]. Hydrolysis of phospholipids in the pre-synaptic membrane is hypothesised to be the primary mode of action of the pre-synaptic PLA_2_ neurotoxins, followed by massive entry of Ca^2+^ into the motor nerve terminal. The increased Ca^2+^ is likely to provide the optimum condition for PLA_2_ activity. These two events, in combination, are likely to lead to exocytosis of the affected nerve terminals, with reduced endocytosis, leading to a depletion of synaptic vesicles in the nerve terminal **[6,7,18,19,21,22]**. In addition, it has been shown that once snake venom PLA_2_ neurotoxins enter the motor nerve terminal, they specifically bind to the mitochondria in the axoplasm, triggering depolarization and a subsequent change of the morphology of the nerve terminal from an elongated shape to a swollen and rounded shape. The impairment of the mitochondrial activity is likely to further damage the motor nerve terminal [7].

We investigated the effect of antivenom on venom-mediated pre- and post-synaptic neurotoxicity and showed that this could be mimicked by washing the neuromuscular preparation with the physiological salt solution, at least for the venom at 3 µg/mL. At 10 µg/mL venom, although both antivenom and washing were equally capable of preventing and reversing post-synaptic effects, only the antivenom was able to prevent or partially prevent the pre-synaptic effects compared to washing. The similar ability to prevent pre-synaptic neurotoxicity, and its effective window of timing by washing and antivenom at 3 µg/mL venom, could be explained as the washing of the bath physically removes the toxins which are in contact with the neuromuscular preparation, preventing further entry of pre-synaptic neurotoxins. The antivenom molecules exert a similar effect by binding with the toxins, thus preventing the toxins further entering the motor nerve terminals. However, both washing and antivenom are unlikely to have an effect on the pre-synaptic neurotoxins that have already initiated their effects on the motor nerve terminals. In experiments with 10 µg/mL venom, the antivenom concentrations were increased corresponding to the venom, which is likely to result in comparably effective removal of toxins (as seen with 3 µg/mL venom) from the neuromuscular junctions due to similar toxin to antibody ratios. However, at 10 µg/mL venom in contrast to 3 µg/mL venom, washing the preparation failed to prevent the pre-synaptic neurotoxicity due to the ineffective physical removal of toxins by washing when the toxin concentrations are too high to be completely washed away.

The lack of effect of antivenom in reversing the already initiated pre-synaptic neurotoxicity has previously been observed clinically as well as experimentally [4,5,6,23,24,25]. Therefore, it was hypothesized that once the pre-synaptic neurotoxins enter the nerve terminals, antibodies or their fragments are unlikely to reverse the pathophysiological process as (1) they are unlikely to enter into the motor nerve terminal and, even if they enter into the motor nerve terminal, (2) they are unlikely to reverse the structural damage caused by the toxins at the motor nerve terminal [4,5,11]. Our study reinforces the above hypothesis. In addition, we have shown that there is still a short period of time in which the antivenom and washing prevent pre-synaptic neurotoxicity. This is likely to be binding or washing of venom that is still in the synaptic cleft, before it becomes irreversibly bound.

The Australian polyvalent antivenom remained effective in at least partially preventing the venom-mediated pre-synaptic neuromuscular block for 15 min after exposing the neuromuscular preparation to the venom. Previously, using the mouse phrenic nerve-hemidiaphragm preparation (i.e., a different skeletal muscle preparation), Australian polyvalent antivenom and a new whole IgG Papuan taipan antivenom were able to at least partially prevent *O. scutellatus* venom mediated neuromuscular block, at the same concentration as in our study, when added to the bath 10 min after the venom [26]. Although the rodent phrenic nerve-hemidiaphragm preparation is unable to distinguish between the pre-and post-synaptic toxins (hence the possible post-synaptic effects of the whole venom-mediated neuromuscular block cannot be excluded), the same study reproduced a similar effect by the two antivenoms when purified taipoxin was used instead of the whole venom.

It appears that there is at least a 10–15 min lag period for the antivenom action in preventing the pre-synaptic neurotoxin-mediated neuromuscular block. In human envenoming, there is a delay following the bite due to the time taken by the toxins to be absorbed from the venom injection site (usually subcutaneous or intramuscular) into the lymphatics and then to reach the motor nerve terminals through the circulation. This means there is likely to be a longer time period in which antivenom remains effective in neutralizing venom prior to it reaching the neuromuscular junction [11]. Acknowledging this time period, in which antivenoms remain effective for pre-synaptic neurotoxicity of snakebite, more targeted therapies for snake pre-synaptic neurotoxicity are being experimentally tested. A recent study showed that a PLA_2_ inhibiter, varespladib (LY315920), inhibits coastal taipan venom-mediated inhibition of indirect twitches of the mouse phrenic nerve-diaphragm preparation, within 30 min post-venom, even after the onset of the decline in twitch responses [27]. Although the mouse phrenic nerve-diaphragm preparation cannot distinguish pre- and post-synaptic effects, it could be assumed that varespladib may have potential in reversing the pre-synaptic neurotoxicity more effectively, compared to antivenom.

Many snakes possess both pre- and post-synaptic neurotoxins in their venoms. The nature of in vitro neuromuscular block (i.e., pre- or post-synaptic) at the different concentrations of the whole venom is likely to depend on the relative abundances of the pre- and post-synaptic neurotoxins in the venom, the binding, potencies and the time taken for the maximum pharmacological effect of the individual neurotoxins in the venom. In general, the time taken for the maximum neuromuscular blocking effect (which corresponds to the time taken for the maximum inhibition of indirect twitches in the neuromuscular preparation) of α-neurotoxins is shorter than pre-synaptic neurotoxins [9,13,19]. In this study, the action of the post-synaptic neurotoxins, at the lower venom concentration, was not observed, and the neuromuscular blocking effect on the chick biventer neuromuscular preparation was solely pre-synaptic in origin. At 10 µg/mL of venom, although both types of toxins likely contributed to the neuromuscular block, only the action of post-synaptic neurotoxins could be observed in the neuromuscular preparation as the response of the chick biventer neuromuscular preparation to ACh and CCh is abolished by post-synaptic neurotoxins (which act faster than the pre-synaptic toxins) masking the effect of the pre-synaptic toxins. The pre-synaptic neuromuscular block caused by 10 µg/mL *O. scutellatus* venom was observed when antivenom was added, or washing commenced, at 30 min, reversing the post-synaptic neuromuscular block caused by the venom. *O. scutellatus* venom contains three short-chain post-synaptic neurotoxins, and no long-chain post-synaptic neurotoxins were isolated from the venom [16,17]. The neuromuscular block caused by short-chain neurotoxins are readily reversible, [9,10] hence explaining the similar reversal effects caused by washing and the antivenom.

## 4. Conclusions

This study demonstrated that in the chick biventer cervicis neuromuscular preparation, *O. scutellatus* produces pre-synaptic predominate neuromuscular block at a concentration of 3 µg/mL and post-synaptic predominate neuromuscular block at a concentration of 10 µg/mL. The post-synaptic effects of the venom could be readily reversed by antivenom even 30 min after the venom was applied. However, there only exists a 10–15 min lag period in which the antivenom can prevent the pre-synaptic neurotoxin-mediated neuromuscular block. In human snake envenoming, this time window is likely to be longer due to the delay in which venom is absorbed, and the time venom takes to reach the neuromuscular junction [4,24,25,28]. Our study also provides evidence that the antivenom is unlikely to reverse events occurring inside the nerve terminal, and most likely acts by binding pre-synaptic toxins before they interfere with neurotransmission inside the motor nerve terminals.

## 5. Materials and Methods

### 5.1. Venoms, Toxins and Antivenoms

Freeze-dried pooled Australian coastal taipan (*O. scutellatus*) venom (Venom Supplies, Tanunda, South Australia) was used for this study. Venom was dissolved in distilled water and stored at −80 °C until required. Taipoxin was isolated and purified from *O. scutellatus* venom using size-exclusion chromatography, following the method described in Barber et al. [29]. Protein quantifications of the venom and taipoxin were carried out using a BCA Protein Assay Kit (Thermo Fisher Scientific, Rockford, IL, USA) as per manufacturer’s instructions. Australian polyvalent antivenom (equine) (Seqirus, Parkville, VIC, Australia; Batch No: 055519001) was used for this study. Australian antivenoms come in liquid form and, according to the manufacturer’s instructions, this vial of polyvalent antivenom contained 12,000 units of *O. scutellatus* antivenom in 48.54 mL.

### 5.2. Chick-Biventer Cervicis Nerve-Muscle Preparation

Male chickens (aged 4–10 days) were humanely killed by exsanguination following CO_2_ inhalation. Biventer cervicis nerve-muscle preparations were dissected and then mounted on wire tissue holders under 1 g resting tension in 25 mL organ baths. Tissues were maintained at 34 °C, bubbled with 95% O_2_ and 5% CO_2_, in physiological salt solution of the following composition (mM): 118.4 NaCl, 4.7 KCl, 1.2 MgSO_4_, 1.2 KH_2_PO_4_, 2.5 CaCl_2_, 25 NaHCO_3_ and 11.1 glucose. Indirect twitches were evoked by stimulating the motor nerve (rate: 0.1 Hz; pulse duration: 0.2 ms) at supramaximal voltage (7–15 V), using an electronic stimulator (ADInstruments, Quincy, MA, USA). Selective stimulation of the nerve was confirmed by the abolishment of twitches with d-tubocurarine (10 µM, Sigma-Aldrich, St. Louis, MO, USA). Tissues were then repeatedly washed with physiological salt solution to restore twitch response to nerve stimulation. Contractile responses of the tissues to exogenous acetylcholine (ACh; HIMedia, Vadhani Industrial Estate, Mumbai, India; 1 mM for 30 s), carbachol (CCh; Sigma-Aldrich, St. Louis, MO, USA; 20 µM for 60 s) and KCl (40 mM for 30 s) were obtained in the absence of nerve stimulation. The preparations were then stimulated for 10 min, before the experiments were commenced. All experiments were run for up to 3 h. At the conclusion of the experiment, ACh, CCh and KCl were re-added as above.

### 5.3. Experimental Design

Initial experiments were carried out to select individual venom and toxin concentrations that consistently abolished indirect twitches. Based on these preliminary studies, venom was used at 1, 3 and 10 µg/mL to identify venom concentrations that resulted in primarily presynaptic neurotoxicity, and the concentrations that result in both pre- and post-synaptic neurotoxicity. The addition of large amounts of antivenom can alter the osmolarity of the physiological salt solution, so antivenom control experiments (i.e., antivenom only) were performed to ensure that the antivenom was not affecting the tissue viability.

To test the ability of the antivenom to arrest or reverse the neuromuscular block, the antivenom was added either 5, 15 or 30 min after venom/toxin. In washing experiments, instead of adding the antivenom, the preparation was washed with the physiological salt solution (flushed the bath and filled with buffer two times) at 5, 15 or 30 min after addition of the venom/toxin.

For all venom experiments, the required antivenom amount was calculated based on the definition that one unit of antivenom neutralizes 10 µg of venom. For all taipoxin experiments, 112 µL of antivenom was used for 0.1 nM toxin in a 25 mL bath, following initial experiments to ensure full neutralization of neurotoxicity was achieved at this quantity when antivenom was added before the toxin.

### 5.4. Data Analysis and Statistics

Indirect twitch responses and responses to exogenous agonists (ACh, CCh and KCl) were measured via a MLT0201 force transducer (ADInstruments Pty Ltd., Bella Vista, NSW, Australia) and recorded on a PowerLab system (ADInstruments Pty Ltd., Bella Vista, NSW, Australia). All twitch and agonist responses were expressed as percentages of their pre-venom/toxin values. A one-way ANOVA was used to compare the responses to exogenous agonists following the administration of venom. All ANOVAs were followed by Tukey’s multiple comparison post-tests. Data are presented in the form of mean with the standard error of the mean (S.E.M.) of three to seven experiments. All statistical analyses and presentation of data were generated using GraphPad Prism 8.0.2 software (GraphPad software Inc., La Jolla, CA, USA). For all statistical tests, *p* < 0.05 was considered statistically significant.

### 5.5. Animal Ethics

All animal experiments used in this study were approved by the Ethics Review Committee of Faculty of Medicine and Allied Sciences, Rajarata University (Approval no: ERC/2018/13, date of approval: 1 July 2018).
